# Novel MIPs-Parabens based SPE Stationary Phases Characterization and Application

**DOI:** 10.3390/molecules24183334

**Published:** 2019-09-13

**Authors:** Angela Tartaglia, Abuzar Kabir, Songul Ulusoy, Halil Ibrahim Ulusoy, Giuseppe Maria Merone, Fabio Savini, Cristian D’Ovidio, Ugo de Grazia, Serena Gabrielli, Fabio Maroni, Pantaleone Bruni, Fausto Croce, Dora Melucci, Kenneth G. Furton, Marcello Locatelli

**Affiliations:** 1 Department of Pharmacy, University of Chieti–Pescara “G. d’Annunzio”, Via dei Vestini 31, 66100 Chieti, Italy; angela.tartaglia@unich.it (A.T.); fabio.maroni@unich.it (F.M.); pantaleonebruni@libero.it (P.B.); fausto.croce@unich.it (F.C.); 2 Department of Chemistry and Biochemistry, International Forensic Research Institute, Florida International University, 11200 SW 8th St, Miami, FL 33199, USA; furtonk@fiu.edu; 3 Department of Chemistry, Faculty of Science, Cumhuriyet University, Sivas 58140, Turkey; sonulusoy@yahoo.com; 4 Department of Analytical Chemistry, Faculty of Pharmacy, Cumhuriyet University, Sivas 58140, Turkey; hiulusoy@yahoo.com; 5 Department of Neuroscience, Imaging and Clinical Sciences, University of Chieti–Pescara “G. d’Annunzio”, Via dei Vestini 31, 66100 Chieti, Italy; giuseppe.merone@unich.it; 6 Pharmatoxicology Laboratory—Hospital “Santo Spirito”, Via Fonte Romana 8, 65124 Pescara, Italy; fabio.savini@ausl.pe.it; 7 Department of Medicine and Aging Sciences, Section of Legal Medicine, University of Chieti–Pescara “G. d’Annunzio”, Via dei Vestini 31, 66100 Chieti, Italy; cristian.dovidio@unich.it; 8 IRCCS Neurological Institute Foundation Carlo Besta, Laboratory of Neurological Biochemistry and Neuropharmacology, Via Celoria 11, 20133 Milan, Italy; ugo.degrazia@istituto-besta.it; 9 Chemical Science Department, School of Science and Technology, University of Camerino, Piazza Cavour 19/f, 62032 Camerino, Italy; serena.gabrielli@unicam.it; 10 Department of Chemistry “G. Ciamician”, University of Bologna, Via Selmi 2, 40126 Bologna, Italy; dora.melucci@unibo.it

**Keywords:** MIPs, parabens, biological matrix, extraction procedure, HPLC-PDA, stationary phase characterization

## Abstract

In this work, the synthesis, characterization, and application of novel parabens imprinted polymers as highly selective solid-phase extraction (SPE) sorbents have been reported. The imprinted polymers were created using sol–gel molecular imprinting process. All the seven parabens were considered herein in order to check the phase selectivity. By means of a validated HPLC-photodiode array detector (PDA) method all seven parabens were resolved in a single chromatographic run of 25 min. These SPE sorbents, *in-house* packed in SPE empty cartridges, were first characterized in terms of extraction capability, breakthrough volume, retention volume, hold-up volume, number of theoretical plates, and retention factor. Finally, the device was applied to a real urine sample to check the method feasibility on a very complex matrix. The new paraben imprinted SPE sorbents, not yet present in the literature, potentially encourage the development of novel molecularly imprinted polymers (MIPs) to enhance the extraction efficiency, and consequently the overall analytical performances, when the trace quantification is required.

## 1. Introduction

Parabens are alkyl esters of *p*-hydroxybenzoic acid, commonly used as antimicrobial agents and preservatives in food products, pharmaceutical preparations, and cosmetic products [1]. This family includes methyl paraben (MPB), ethyl paraben (EPB), iso-propyl paraben (iPPB), propyl paraben (PPB), iso-butyl paraben (iBPB), butyl paraben (BPB), and benzyl paraben (BzPB). Their molecular formula, molecular weight, and chemical structure are shown in Table 1. Low cost, broad spectrum of activity, and chemical and thermal stability explain their widespread application compared to other alternatives [2].

Humans may be exposed to parabens through inhalation, dermal contact, and ingestion. Parabens have been considered low toxicity compounds; nevertheless, there is growing evidence of their implication as endocrine disruption compounds (EDCs), i.e., products with negative effects on endocrine system due to their interaction with production, release, transport, metabolism, or elimination of hormones. Many studies describe the toxic effects of parabens such as obesity, diabetes, breast cancer and problems in reproductive system [3].

According to the European Union (EU) and the United States Food and Drug Administration (US FDA), the concentration of parabens should not exceed 0.4% for a single paraben and 0.8% for a mixture of parabens in the cosmetics product; the concentration in drugs must be less than 0.1% and 0.3% for individual paraben and for parabens mixture, respectively [4].

During the last years, new methods have been developed for the determination of parabens in different matrices. Due to the low concentrations of these compounds, the sample preparation represents the critical step for the isolation and analysis of these target molecules. Usually, common extraction techniques were used for pre-concentrating parabens, such as solid-phase extraction (SPE) [5,6,7,8] and solid-phase microextraction (SPME) [9]. For SPE, different materials are available for extraction, preconcentration, and sample cleaning. Such sorbents used in solid-phase extraction can be divided into three main groups: silica sorbents, polymeric sorbents, and activated or graphitized carbon. Analytical parameters such as selectivity, affinity, and extraction capacity are closely associated with the sorbent used and, consequently, various SPE materials have been developed to replace the classic ones and to increase the extractive selectivity. An example is represented by molecularly imprinted polymers (MIPs) used as SPE sorbent [1].

**Figure 1:**
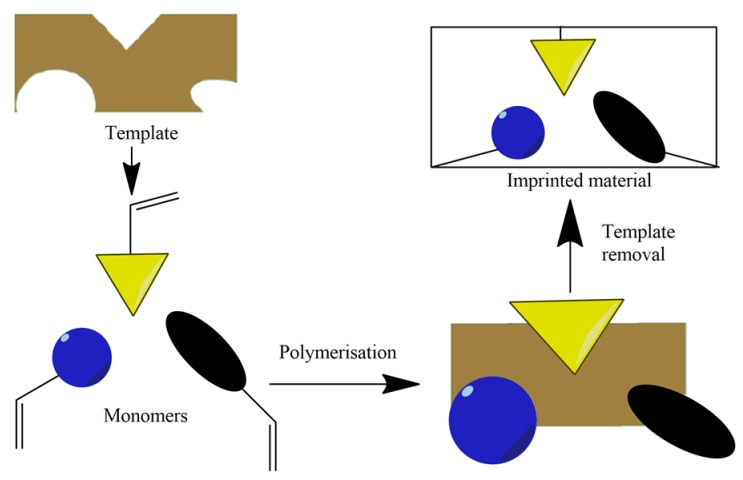
Synthesis of molecularly imprinted polymers (MIPs).

MIPs are formed by fixing a molecule (called a “template”) on the polymer; the molecule is extracted afterward, leaving complementary cavities behind [10]. Molecular imprinting is obtained by polymerization of monomers in the presence of template molecules (Figure 1). The imprinting template should be stable under polymerization reaction. The link between template and monomer must be strong to form the pocket but also weak enough to be able to remove the template at the end. Usually, the monomer and template are in 4:1 molar ratio. The most frequently used monomers are methacrylic acid and 4-vinylpyridine [11].

There are different methods used to perform molecular imprinting: covalent imprinting, non-covalent imprinting, and semi-covalent imprinting. In covalent imprinting, the template and monomer are linked by covalent bonds; after polymerization, the template is removed from the polymer through the cleavage of covalent bond. The template is replaced from target analytes that bind forming the same covalent links. In non-covalent imprinting, template and monomer are linked by non-covalent interactions such as hydrogen bonding, ionic interactions, π–π interactions, and van der Waals forces. After polymerization, the template is removed, and the target analytes bind the polymer via same non-covalent interactions. This technology is the most popular for the synthesis of MIP. In semi-covalent approach, the template is linked covalently to the monomer but is rebinding by non-covalent interactions [12].

Among several MIP synthesis approaches, sol–gel synthesis process for creating MIPs has drawn tremendous attention due to ruggedness of the polymers originated from hybrid inorganic–organic components, high thermal and solvent stability, and ability to maintain imprinted cavities even after multiple use as well as their characteristic high imprinting factor (IF) compared to organic MIP synthesis approach. The success of sol–gel based MIP synthesis primarily depends on the judicious selection of sol–gel functional precursor(s), networking precursor, and the reaction conditions including the catalysts. In the current study, 3-aminopropyl triethoxysilane and phenyltriethoxysilane were used as the sol–gel functional polymers to provide hydrogen bonding and π–π interactions toward the template molecules, respectively. Tetramethyl orthosilicate was used as the networking sol–gel precursor, whose primary role was to rigidly hold the template-sol–gel functional precursors complexes in the sol–gel 3D network [13,14]. The detailed MIPs synthesis process is described in Section 3.5.

Recently, molecularly imprinted polymers have aroused great interest and have been used in different fields, particularly for analytes extraction in complex samples. In the literature, just few published papers report the development of MIP-propyl paraben based and/or based on a limited selection of the above-cited molecules. Furthermore, such methods consider only environmental sample matrices. In the current study, following our research on innovative (micro)extraction procedures [15,16,17,18,19,20,21,22,23,24,25], different molecularly imprinted sorbent materials were studied for the extraction of most common parabens, including MPB, EPB, iPPB, PPB, iBPB, BPB, and BzPB. These sorbent phases were newly synthesized in our laboratory and herein tested in order to compare them in terms of major analytical parameters such as breakthrough volume (*V_B_*), retention volume (*V_R_*), hold-up volume (*V_M_*), retention factor (*k*), and theoretical plates number (*N*). Furthermore, studies on selectivity have been carried out in this work in order to obtain a full characterization for these new sorbent materials when applied on real urine samples.

## 2. Results and Discussion

### 2.1. Experimental Determination of the Breakthrough Volume

The extraction capacity in the SPE depends on many factors such as the analyte retention capacity, the loaded sample volume, the conditioning solvents type, and volumes [26].

Nowadays, many new materials are available as sorbent material for SPE. To understand the extraction mechanism of these materials and to improve the extraction efficiency, it is important to characterize them. In SPE, the most important parameters that need to be calculated are breakthrough volume (*V_B_*), retention volume (*V_R_)*, hold-up volume (*V_M_*), retention factor (*k*), and theoretical plates number (*N*). For the determination of breakthrough volume, a solution containing the target analytes is continually applied to the SPE cartridge containing the sorbent material; breakthrough occurs when the capacity of sorbent has been exhausted [15,26,27].

**Figure 2:**
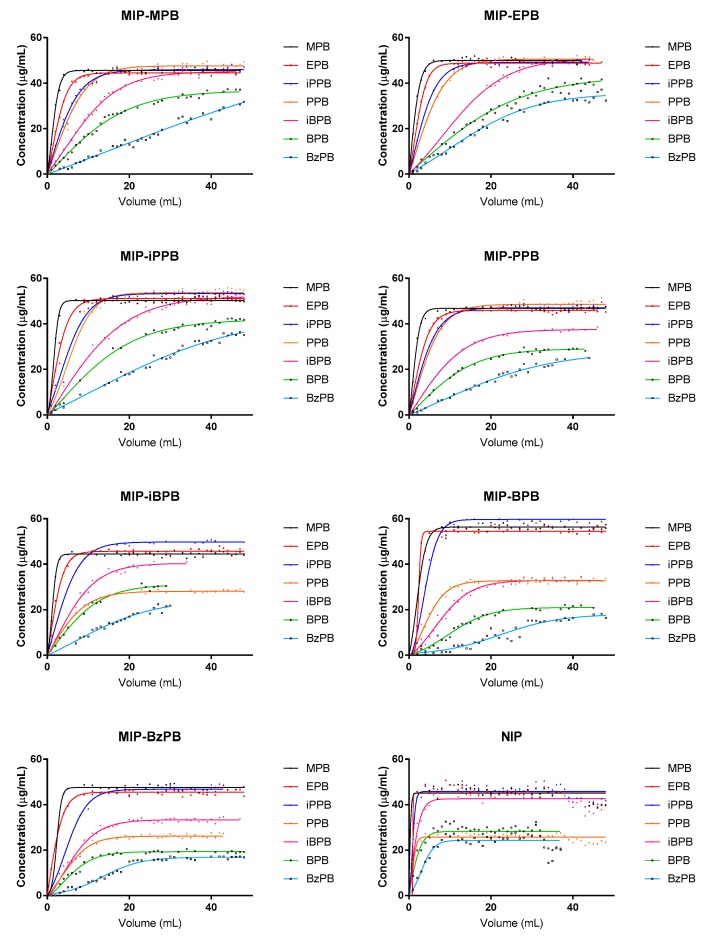
Boltzmann’s functions determined for parabens on the different MIPs sorbent.

The experimental curves (concentration vs. solution volume) were fitted by means of Boltzmann’s function, and the regression parameters were used in calculating the parameters (breakthrough volume, retention volume, hold-up volume, retention factor, and theoretical plates number) for each considered stationary phases loaded on SPE cartridges [15,26,27]. Figure 2 reports the Boltzmann’s functions by plotting the concentration of the analyte against the aliquot volumes passed through the sorbent for each type of MIPs and for all tested analytes. These graphs represent the dependence of parabens concentrations on successive 50 mL volume loading of aqueous samples. Table 2 reports the breakthrough volume (*V_B_*), retention volume (*V_R_*), hold-up volume (*V_M_*), retention factor (*k*), and theoretical plates number (*N*) values calculated by using the proposed mathematical approach [15,26,27]. From the results presented in Table 2, it is evident that, as expected, the MIPs have a higher affinity for the molecule than the non-imprinted polymers (NIPs) and the polymer imprinted with BzPB has the highest breakthrough volumes, showing the highest capacity in trapping the analytes. 

As reported by Bacalum et al. [27], the concentration of some compounds has different values at infinite time when passing through different MIP cartridges, and this finding could be explained by a change of interaction between analyte and the sorbent (e.g., two distinct adsorption profiles: the first herein observed, the second at higher sample volumes). Additionally, as reported by Bielica-Daszkiewicz [26] and Bacalum [27], it can be possible to observe curves where the maximum concentration used to test the sorbents not reached. 

Furthermore, the curves obtained for the NIP show a shape change at very low volumes, highlighting that no retention mechanisms occur. Only for BzPB could be observed a little change, probably related to the different interactions, which could occur in relation to the chemical structure. In fact, in the presence of this analyte, larger breakthrough volume values are observed in all types of prepared MIPs, including NIPs. In this case, not only the interactions linked to the common basic structure for all parabens could be present but also there could be greater retention probably related to the second aromatic system (with related π–π interactions).

### 2.2. Selectivity Study of Molecularly Imprinted Polymers

Competitive adsorptions of methyl paraben, ethyl paraben, propyl paraben, *iso*-propyl paraben, propyl paraben, *iso*-butyl paraben, butyl paraben, and benzyl paraben were evaluated using imprinted and non-imprinted polymer. The Boltzmann’s functions calculated for single paraben compared to all different molecular imprinted polymers herein tested, including NIP, were reported in Supplementary Materials, Section S.1, Figure S.1.1.

Furthermore, the selectivity that each paraben showed in its molecularly imprinted polymer compared with other imprinted polymers has been evaluated. The deviations could be related to the unselective interactions between the target analyte and the MIP phase. Table 3 shows data from the linear regression carried out to obtain comparative data of each single paraben in the various imprinted polymers, while in Supplementary Materials, the Figure S.1.2 reports the graphs for the single parabens. The values obtained for each paraben in the reference polymer have been placed on the *x*-axis, while on the *y*-axis the data relating to the single parabens in the polymers imprinted with different template are placed. This comparison was carried out in order to better highlight how the imprinted polymers are selective toward the single parabens with respect to other molecules with similar structure. By means of the obtained results, the imprinted polymers show a limited selectivity toward the single paraben, as can be observed from calculated slope values (nearest to 1 for all parabens). In particular, the imprinted polymers with PPB, iBPB, BPB, and BzPB template result more selective toward single parabens compared with MPB, EPB, and iPPB-imprinted polymers.

However, some anomalies related to the PPB, i-BPB; BPB, and BzPB (chemical structures reported in Supplementary Materials, Figure S.1.3) have been found in MIP-PPB and MIP-BPB. MIP-PPB shows low retention capacity toward iBPB and it could be related to the chemical structure (-CH_3_ instead -H). The –CH_3_ group shows a little higher steric hindrance compared to only -H and this may partly limit the retention of this analyte for a still relatively small MIP cavity. This element can also be justified by the fact that, as shown in Table 3, the slope values are >1 for small parabens when the MIP was obtained with larger parabens as template. In addition, MIP-BPB had low retention capacity for BzPB; this is probably related to aromatic group in chemical structure of BzPB that increase the steric hindrance and not allow to a complete fit with the interaction folder.

For NIP, all values reported in Table 3 were below the unit, demonstrating that this non-imprinted polymer shows no selectivity against the considered analytes. As also highlighted in Table 2, NIP shows low breakthrough volume (mL) values in respect all the other MIPs. Furthermore, in Table 2 it was not possible to evaluate several parameters for the NIP as the Boltzmann’s functions reported floating point errors.

From the analyses carried out, it appears evident that most of the selectivity of the phases is linked to the structure common to all the parabens (slope values reported in Table 3 next to unity) and that this is maximized when the same analyte is evaluated used as a different template (unitary slope). When the binding site is similar in size to the analyte, phase selectivity is not observed and interactions with the common paraben structure prevail. Similarly, if the site is larger, it is observed that structurally similar (but smaller in size) analytes are mostly retained (slope greater than 1).

For MPB the values are all close to the unit independently of the MIP. Similarly, similar behavior is observed for EPB and iPPB. Only from the PPB a distinction is made between selectivity based on the slope of the curves with values close to 1 for analytes of smaller dimensions and different from the unit (<1) for larger structures.

### 2.3. Real Sample Analysis

This procedure was applied to real urine sample from a healthy donor, who did not voluntarily take parabens or products containing parabens for analysis. The real sample was derived from our previous project [4], where only EPB was found in one urine real sample (as reported in Section 3.3). The urine sample, stored at −20 °C before analysis, was extracted following the MIP-SPE procedure reported in Section 3.7, and subsequently the eluate was injected in HPLC-PDA system. Using the MIP-EPB as sorbent in the SPE procedure, an improvement of *S/N* ratio of 3–5 times has been observed compared with previously obtained ratio [4], confirming the sensibility of imprinting polymers for this class of compounds. To fully compare fabric phase sorptive extraction-HPLC-PDA (FPSE-HPLC-PDA) vs. MIP-SPE-HPLC-PDA, a partial validation for MIP-SPE based procedure would be necessary, and then to compare the results by mean of statistical approach. From the point of view of the comparison between these 2 configurations, it would, however, be correct to provide for the validation of the new MIP-SPE-HPLC-PDA configuration (in order to have the best and optimized performances) and subsequently it can be applied a *t*-test.

### 2.4. MIPs Characterization

The evaluation of the chemical–physical characteristics of the created MIPs is reported in the Supplementary Materials, Section S.1.4. From the data obtained through Fourier transform infrared spectroscopy (FTIR), differential thermal analysis (DTA), and thermogravimetric (TGA) analysis it is possible to highlight the reproducibility of the synthesis process using different templates.

## 3. Materials and Methods

### 3.1. Chemicals, Solvents, and Devices

The International Forensic Research Institute, Department of Chemistry and Biochemistry, Florida International University (Miami, FL, USA) provided all parabens chemical standards (methyl paraben, ethyl paraben, propyl paraben, isopropyl paraben, butyl paraben, isobutyl paraben, and benzyl paraben) and all molecularly imprinted polymers.

Sodium phosphate monobasic, sodium phosphate dibasic (>99% purity grade), and phosphoric acid were obtained from Sigma-Aldrich (Milan, Italy). Acetonitrile and methanol (HPLC-grade) were purchased from Honeywell (New Jersey, USA) and were used without further purification. Deionized water (18.2 MΩ-cm at 25 °C) was generated by a Millipore MilliQ Plus water (Millipore Bedford Corp., Bedford, MA, USA). GraphPad Prism v.4 was used for the statistical analysis of experimental data.

### 3.2. Stock Solution and Working Solution Preparation

Stock solutions of chemical standards were prepared in methanol at the concentration of 1 mg/mL. The working solutions were prepared by dilution of stock solution in Milli-Q water and stored at 4 °C. The resulting samples were used to evaluate the enrichment factors, breakthrough volume, retention volume, hold-up volume, retention factor, and theoretical plates number.

### 3.3. Human Urine Sample Collection, Storage, and Preparation

Human urine sample was collected from a healthy volunteer informed about the nature of the study, who did not voluntarily take parabens or products containing parabens for analysis purposes. This urine sample is the same (a second aliquot) analyzed in the previous study [4], stored at −20 °C before analysis.

### 3.4. Apparatus and Chromatographic Conditions

The method has been described and validated by Tartaglia et al. [4]. Briefly, analyses were performed using an HPLC Thermo Fisher Scientific liquid chromatography system (Model: Spectra System P2000) coupled to a photodiode array detector (PDA) Model: Spectra System UV6000LP. Mobile phase was directly on-line degassed by using a Spectra System SCM1000 (Thermo Fisher Scientific, Waltham, MA, USA). Excalibur v.2.0 software (Thermo Fisher Scientific, Waltham, MA, USA) was used to collect and analyze data. Spherisorb C18 (15 cm × 4.6 mm, 5 µm) was used to resolve all parabens; the column was thermostated at 27 °C (± 1 °C) using a Jetstream2 Plus column oven during the analysis. The chromatographic separation was conducted in isocratic elution using phosphate buffer (28 mM, pH = 2.5) as solvent A and methanol as solvent B in volume percentages of 55 and 45, respectively. The flow rate was set at 1 mL/min. All the compounds were detected at the maximum wavelengths of 257 nm with retention time of 3.97, 6.00, 8.83, 10.43, 18.37, 19.75, and 22.33 min (for MPB, EPB, iPPB, PPB, iBPB, BPB, and BzPB, respectively). 

### 3.5. Synthesis of Novel MIPs-Parabens and NIP

Preparation of parabens imprinted polymers involve several distinct steps: *(i)* complexation of parabens with sol–gel functional precursors; *(ii)* hydrolysis of the sol–gel cross-linking reagent; *(iii)* condensation of hydrolyzed sol–gel cross-linking reagent in presence of paraben-functional precursor complex; *(iv)* removal of paraben templates from paraben imprinted sorbents; and *(v)* synthesis of non-imprinted polymer (NIP) sorbents.

#### 3.5.1. Complexation of Parabens with Sol–Gel Functional Precursors

The complexation of individual paraben with sol–gel precursors is a spontaneous, self-assembling process directed by intermolecular interactions, e.g., hydrogen bonding, π–π interactions, and van der Waals force between the paraben template and sol–gel functional precursors. To achieve superior specificity, two sol–gel precursors phenyltriethoxysilane (PTES) and 3-aminopropyl triethoxysilane (3-APTES) were employed in the complexation process. The complexation was carried out by sequentially adding the paraben template:ethanol:PTES:3-APTES in a 50-mL centrifuge tube at a molar ratio 1:80:3:3, respectively. The mixture was vortexed vigorously after adding each ingredient. Subsequently, the solution was sonicated for 30 min. The mixture was then incubated at room temperature for 6 h so that the sol–gel functional precursors self-assembled themselves around the template molecules by hydrogen bonding and π–π interactions.

#### 3.5.2. Hydrolysis of Sol–Gel Cross-Linking Reagent

Tetramethyl orthosilicate (TMOS) was used as the sol–gel cross-linking reagent. Four methoxy functional groups connected to the central silica atom must be hydrolyzed first so that they can undergo polycondensation to build the inorganic silica network. Hydrolysis of TMOS was carried under acidic condition using trifluoroacetic acid (TFA) as the acid catalyst. The molar ratio of the template:TMOS:ethanol:TFA:water was maintained at 1:15:45:0.1:55, respectively. The mixture was prepared in a 50-mL centrifuge tube by adding individual ingredient, vortexing for 5 min and finally sonicating for 30 min. The sol solution was kept in a silicon bath at 50 °C for 12 h to ensure complete hydrolysis of TMOS in presence of the acid catalyst.

#### 3.5.3. Condensation of Hydrolyzed Sol–Gel Cross-Linking Reagent in Presence of the Paraben-Functional Precursors Complex

To create paraben imprinted polymers, paraben sol–gel functional precursors complexes are added to hydrolyzed sol solution in droplets under continuous stirring on a magnetic stirrer. During this process, paraben complexes randomly orient themselves within the growing sol–gel network with minimal steric hindrance and subsequently become frozen with the 3D sol–gel network. In order to complete and expedite the network formation process via condensation, the sol–gel polymer was kept in a silicon oil bath at 50 °C for 24 h. During this residence time in the oil bath, aging and ripening of the sol–gel network occurs, leading to a robust, highly porous silica network with trapped solvent, templates, and unreacted sol solution ingredients.

#### 3.5.4. Removal of Paraben Templates from the Imprinted Polymers

Quantitative and exhaustive removal of paraben templates from the imprinted polymer is one of the most important and challenging tasks in the creation of parabens imprinted polymers. Successful removal of the templates leave a nanocavity complimentary to the size, shape, and functionality of the template molecules and creates a highly specific synthetic receptor site, extremely affinitive towards the template molecules. To remove the templates, the paraben imprinted sol–gel polymers were first dried at 100 °C in a vacuum drier for 48 h. The dried MIPs are then crushed and ground in a mortar into fine particles (~50 µm) and subsequently subjected to accelerated solvent extraction at 150 °C and 1500 psi for 30 min using methanol as the extraction solvent. Additionally, during the analyses for breakthrough volume curves, no parabens release was observed in the conditioning steps, highlighting that the phases were completely cleaned during accelerated solvent extraction (ASE) process.

#### 3.5.5. Synthesis of Non-Imprinted Polymers

The success of molecular imprinting is often evaluated by comparing the adsorption of the template molecules on the imprinted polymer bed over a non-imprinted polymer (NIP) bed under identical conditions. Nonspecific adsorption of both the MIP and NIP can be easily estimated by exposing the known mass of the polymers into a known volume of an aqueous solution containing a known concentration of the template molecules. As such, paraben non-imprinted polymers were synthesized in parallel to the MIPs using identical process and sol solution with the only exception that no paraben templates were added in the NIP materials.

### 3.6. Preparation of MIP–SPE Media

A frit was placed on the bottom of an empty SPE cartridge (1 mL in total volume), which was employed as the MIP–SPE column. The SPE cartridges used in this study were in-house packed with 30 mg of parabens-MIPs (the stationary phase synthesized as previously reported in Section 3.5). Then, another frit was placed on the top of the cartridge.

### 3.7. Molecularly Imprinted Polymer–Solid Phase Extraction Procedure

For the breakthrough curve construction, SPE experiment was carried out as follows: 50 mL of solution at 50 µg/mL was introduced into SPE sorbent (1 mL per time), conditioned with methanol (1 mL) and water (1 mL). Each sample aliquot (1 mL) was collected in a separate vial and analyzed by HPLC-PDA to measure the concentration of analyte in each aliquot of sample. Breakthrough curves were determined as the relationship between concentration after extraction and the total volume passed through the sorbent. 

For the analysis of the urine sample, the SPE cartridge loaded with molecularly imprinted polymer was conditioned by 1 mL of methanol and followed 1 mL of Milli-Q water. 1 mL of sample solution was passed through the cartridge at a flow of 1 mL/min. Subsequently, the cartridge was consecutively rinsed with 2 mL of water, and 3 mL of MeOH was used as elution solvent. Elutes (20 μL) are then injected in HPLC-PDA system.

## 4. Conclusions

A novel, MIP sorbent based on seven parabens template were synthesized. The new MIP-SPE method coupled with HPLC-PDA analysis was preliminarily tested for the determination of parabens in human urine, confirming that molecular imprinting technology represents a valid strategy for the synthesis of new selective extraction materials. All molecularly imprinted polymers have been characterized in terms of extraction capability, breakthrough volume, retention volume, hold-up volume, number of theoretical plates, and retention factor.

Furthermore, selectivity studies have been performed, comparing the extraction efficiency of each molecularly imprinted polymer for all the parabens tested in this study. Comparing the calculated parameters, the imprinted polymer does not show marked selectivity for every single paraben, but they provided “*class-specific*” interactions. Following this work, a polymer based on a mixture of individual MIPs for the extraction of these compounds from complex matrices could be developed. Greater efforts are therefore needed in future studies to obtain extractive materials based on imprinted polymers with increased selectivity and specificity. 

## Figures and Tables

**Table 1:** General chemical–physical characteristics of parabens and their chemical structures.

	Molecular Formula	Molecular Weight	Chemical Structure
**MPB**	C_8_H_8_O_3_	152.15 Da	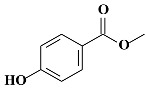
**EPB**	C_9_H_10_O_3_	166.17 Da	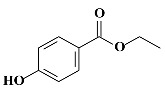
**iPPB**	C_10_H_12_O_3_	180.20 Da	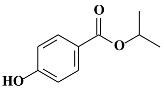
**PPB**	C_10_H_11_O_3_	180.20 Da	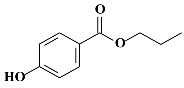
**iBPB**	C_11_H_14_O_3_	194.23 Da	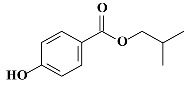
**BPB**	C_11_H_14_O_3_	194.23 Da	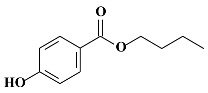
**BzPB**	C_14_H_12_O_3_	228.25 Da	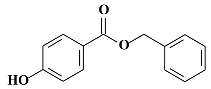

**Table 2:** Parameters determined for the analytes on different MIP sorbent at the concentration of 50 µg/mL.

Sorbent	Analyte	Breakthrough Volume (mL)	Hold-Up Volume (mL)	Retention Volume (mL)	Retention Factor (k)	Theoretical Plates (N)
MIP-MPB	Methyl paraben	2.19	6.78	1.1	2	5.18
	Ethyl paraben	2.1	11.81	1.06	2	10.15
	i-Propyl paraben	0.13	19.85	0.05	0	431.06
	Propyl paraben	2.78	23.93	1.37	2	16.51
	i-Butyl paraben	8.54	41.07	4.21	2	8.75
	Butyl paraben	0.91	50.47	0.53	5	94.1
	Benzyl paraben	3.41	273.36	1.57	1	173.12
MIP-EPB	Methyl paraben	0.53	6.69	0.27	2	24.17
	Ethyl paraben	2.51	10.13	1.26	2	7.07
	i-Propyl paraben	0.83	16.61	0.44	2.65	37.03
	Propyl paraben	1.84	22.22	0.92	2	23.23
	i-Butyl paraben	16.61	54.07	8.21	2	5.58
	Butyl paraben	3.54	69.81	1.59	1	42.99
	Benzyl paraben	20.46	70.82	10	2	6.08
MIP-iPPB	Methyl paraben	3.04	6	1.55	2	2.88
	Ethyl paraben	0.96	12.11	0.54	4	21.36
	i-Propyl paraben	6.13	22.64	2.97	1.64	6.63
	Propyl paraben	9.58	24.69	4.73	2	4.22
	i-Butyl paraben	1.96	47.96	1.15	5	40.6
	Butyl paraben	3.3	53.73	1.77	3	29.37
	Benzyl paraben	22.72	115.06	10.95	2	9.51
MIP-PPB	Methyl paraben	1.82	6.35	0.92	2	5.89
	Ethyl paraben	1.01	12.29	0.51	2	23.06
	i-Propyl paraben	0.56	17.24	0.29	2.59	58.4
	Propyl paraben	1.54	19.89	0.8	2	23.95
	i-Butyl paraben	0.84	34.91	0.46	3	74.51
	Butyl paraben	1.6	40.73	0.85	3	46.9
	Benzyl paraben	14.96	84.68	7.44	2	10.39
MIP-iBPB	Methyl paraben	2.42	5.13	1.21	2	3.25
	Ethyl paraben	1.06	9.85	0.56	3	16.74
	i-Propyl paraben	2.01	19.55	1	1.99	18.5
	Propyl paraben	0.41	23.83	0.22	3	105.23
	i-Butyl paraben	3.16	28.74	1.63	2	16.65
	Butyl paraben	2.14	33.26	1.06	2	30.38
	Benzyl paraben	13.18	62.6	6.63	2	8.44
MIP-BPB	Methyl paraben	3.8	9.39	1.9	2	3.96
	Ethyl paraben	4.18	5.59	2.35	4	1.37
	i-Propyl paraben	6.56	14.92	3.54	3.16	3.21
	Propyl paraben	5.56	19.85	2.99	3	5.63
	i-Butyl paraben	12.17	33.06	6.31	2	4.24
	Butyl paraben	19.14	43.04	9.52	2	3.52
	Benzyl paraben	54.34	88.58	22.22	1	2.99
MIP-BzPB	Methyl paraben	4.63	7.39	2.31	2	2.2
	Ethyl paraben	0.17	9.85	0.08	1	125.6
	i-Propyl paraben	8.58	21.77	4.28	1.96	4.09
	Propyl paraben	7.16	23.74	3.57	2	5.66
	i-Butyl paraben	6.62	27.27	3.55	3	6.67
	Butyl paraben	6.85	25.19	3.71	3	5.79
	Benzyl paraben	30.46	52.47	13.42	1	2.91
NIP	Methyl paraben	-	-	-	-	-
	Ethyl paraben	-	-	-	-	-
	i-Propyl paraben	1.71	3.61	0.86	1.98	3.22
	Propyl paraben	1.84	4.06	0.92	2	3.41
	i-Butyl paraben	0.11	6.69	0.06	3	109.55
	Butyl paraben	-	7.53	-	3	-
	Benzyl paraben	6.3	13.55	3.02	2	3.49

**Table 3:** Linear regression data.

Analyte	Sorbent	Slope	Y-Intercept	X-Intercept	1/Slope
MPB	MIP-MPB	1	0	0	1
	MIP-EPB	1.046 ± 0.025	1.85 ± 1.05	−1.77	0.96
	MIP-iPPB	1.134 ± 0.023	−1.29 ± 0.98	1.14	0.88
	MIP-PPB	0.999 ± 0.025	1.2 ± 1.1	−1.2	1.0
	MIP-iBPB	0.927 ± 0.033	2.6 ± 1.4	−2.8	1.1
	MIP-BPB	1.295 ± 0.037	−2.7 ± 1.6	2.07	0.77
	MIP-BzPB	1.039 ± 0.026	0.1.145	−0.12	0.96
	NIP	0.972 ± 0.064	0.2.82	−0.25	1.0
EPB	MIP-MPB	0.944 ± 0.027	−1.7 ± 1.1	1.8	1.1
	MIP-EPB	1	0	0	1
	MIP-iPPB	1.098 ± 0.027	−2.7 ± 1.2	2.50	0.91
	MIP-PPB	0.952 ± 0.026	−0.45 ± 1.2	0.48	1.05
	MIP-iBPB	0.952 ± 0.026	−0.45 ± 1.2	0.48	1.05
	MIP-BPB	1.214 ± 0.075	−3.3.305	2.75	0.82
	MIP-BzPB	0.898 ± 0.019	1.53 ± 0.84	−1.7	1.1
	NIP	0.604 ± 0.090	16.4 ± 4.1	−27.1	1.7
iPPB	MIP-MPB	0.803 ± 0.029	3.2 ± 1.4	−4.0	1.2
	MIP-EPB	0.907 ± 0.015	1.73 ± 0.69	−1.9	1.1
	MIP-iPPB	1	0	0	1
	MIP-PPB	0.853 ± 0.018	1.94 ± 0.89	−2.3	1.2
	MIP-iBPB	0.915 ± 0.014	1.00 ± 0.68	−1.1	1.1
	MIP-BPB	1.115 ± 0.050	1.2.412	−1.09	0.90
	MIP-BzPB	0.875 ± 0.029	0.06 ± 1.4	−0.07	1.1
	NIP	0.387 ± 0.085	25.6 ± 4.1	−66.2	2.6
PPB	MIP-MPB	1.011 ± 0.035	−2.2 ± 1.5	2.18	0.99
	MIP-EPB	1.045 ± 0.026	−0.5 ± 1.1	0.45	0.96
	MIP-iPPB	1.13 ± 0.022	−1.3 ± 1.0	1.14	0.88
	MIP-PPB	1	0	0	1
	MIP-iBPB	0.581 ± 0.017	−0.50 ± 0.78	0.85	1.7
	MIP-BPB	0.722 ± 0.021	−2.41 ± 0.96	3.3	1.4
	MIP-BzPB	0.541 ± 0.025	−0.25 ± 1.2	0.5	1.8
	NIP	0.282 ± 0.051	12.1 ± 2.3	−42.8	3.5
iBPB	MIP-MPB	1.022 ± 0.061	−1.7 ± 1.9	1.62	0.98
	MIP-EPB	1.021 ± 0.031	0.08 ± 1.00	−0.08	0.98
	MIP-iPPB	1.16 ± 0.11	−3.3.91	2.87	0.86
	MIP-PPB	0.884 ± 0.025	−0.25 ± 0.64	0.3	1.1
	MIP-iBPB	1	0	0	1
	MIP-BPB	0.827 ± 0.047	−2.2 ± 1.4	2.7	1.2
	MIP-BzPB	0.382 ± 0.052	−2.7 ± 1.7	7.2	2.6
	NIP	0.81 ± 0.12	15.1 ± 4.0	−18.7	1.2
BPB	MIP-MPB	1.521 ± 0.061	2.23 ± 0.81	−1.46	0.66
	MIP-EPB	1.723 ± 0.087	−0.3 ± 1.4	0.19	0.58
	MIP-iPPB	1.795 ± 0.060	1.3 ± 1.0	−0.72	0.56
	MIP-PPB	1.233 ± 0.056	2.97 ± 0.82	−2.41	0.81
	MIP-iBPB	1.46 ± 0.14	4.4 ± 1.4	−3.01	0.69
	MIP-BPB	1	0	0	1
	MIP-BzPB	0.919 ± 0.098	3.3 ± 1.1	−3.6	1.1
	NIP	0.55 ± 0.17	19.9 ± 2.4	−35.9	1.8
BzPB	MIP-MPB	1.40 ± 0.13	−1.1.645	0.76	0.72
	MIP-EPB	1.879 ± 0.061	0.69 ± 0.73	−0.37	0.53
	MIP-iPPB	1.78 ± 0.13	0.3 ± 1.8	−0.17	0.56
	MIP-PPB	1.220 ± 0.067	0.92 ± 0.80	−0.75	0.82
	MIP-iBPB	1.114 ± 0.064	2.66 ± 0.74	−2.39	0.90
	MIP-BPB	0.895 ± 0.081	−1.52 ± 0.98	1.7	1.1
	MIP-BzPB	1	0	0	1
	NIP	0.75 ± 0.18	13.8 ± 2.0	−18.5	1.3

## Supplementary Materials

The following are available online at https://www.mdpi.com/1420-3049/24/18/3334/s1, Figure S.1.1. Boltzmann’s functions for single parabens., Figure S.1.2. Selectivity of each paraben., Figure S.1.3. Chemical structures of **a.** Propyl paraben; **b.** *iso*–Butyl paraben; **c.** Butyl paraben; **d.** Benzyl paraben. All the axes report the concentration values (µg/mL).

## References

[1] Piao C., Chen L., Wang A. (2014). A review of the extraction and chromatographic determination methods for the analysis of parabens. J. Chromatogr. B.

[2] Ocaña-González J.A., Villar-Navarro M., Ramos-Payán M., Fernández-Torres R., Bello-López M.A. (2015). New developments in the extraction and determination of parabens in cosmetics and environmental samples. A review. Anal. Chim. Acta.

[3] Giulivo M., De Alda M.L., Capri E., Barceló D. (2016). Human exposure to endocrine e disrupting compounds: Their role in reproductive systems, metabolic syndrome and breast cancer. A review. Environ. Res..

[4] Tartaglia A., Kabir A., Ulusoy S., Sperandio E., Piccolantonio S., Ulusoy H.I., Furton K.G., Locatelli M. (2019). FPSE-HPLC-PDA analysis of seven paraben residues in human whole blood, plasma, and urine. J. Chromatogr. B.

[5] Ye X., Kuklenyik Z., Bishop A.M., Needham L.L., Calafat A.M. (2006). Quantification of the urinary concentrations of parabens in humans by on-line solid phase extraction-high performance liquid chromatography–isotope dilution tandem mass spectrometry. J. Chromatogr. B.

[6] Gonzalez-Marino I., Quintana J.B., Rodrıguez I., Cela R. (2009). Simultaneous determination of parabens, triclosan and triclocarban in water by liquid chromatography/electrospray ionization tandem mass spectrometry. Mass Spectrom..

[7] Blanco E., Casais M.d.C., Mejuto M.d.C., Cela R. (2009). Combination of off-line solid-phase extraction and on-column sample stacking for sensitive determination of parabens and p-hydroxybenzoic acid in waters by non-aqueous capillary electrophoresis. Anal. Chim. Acta.

[8] Zotou A., Sakla I., Tzanavaras P.D. (2007). LC-determination of five paraben preservatives in saliva and toothpaste samples using UV detection and a short monolithic column. J. Pharm. Biomed. Anal..

[9] Regueiro J., Becerril E., Garcia-Jares C., Llompart M. (2009). Trace analysis of parabens, triclosan and related chlorophenols in water by headspace solid-phase microextraction with in situ derivatization and gas chromatography–tandem mass spectrometry. J. Chromatogr. A.

[10] Sanagi M.M., Salleh S., Ibrahim W.A.W., Naim A.A., Hermawan D., Miskam M., Hussain I., Aboul-Enein H.Y. (2012). Molecularly imprinted polymer solid-phase extraction for the analysis of organophosphorus pesticides in fruit samples. J. Food Compos. Anal..

[11] Li G., Row K.H. (2018). Recent applications of molecularly imprinted polymers (MIPs) on micro-extraction techniques. Sep. Purif. Rev..

[12] Chen L., Xu S., Li J. (2011). Recent advances in molecular imprinting technology: Current status, challenges and highlighted applications. Chem. Soc. Rev..

[13] Lofgreen J.E., Ozin G.A. (2014). Controlling morphology and porosity to improve performance of molecularly imprinted sol-gel silica. Chem. Soc. Rev..

[14] Diaz-Garcia M.E., Laino R.B. (2005). Molecular Imprinting in sol-gel materials: Recent developments and applications. Microchim. Acta.

[15] Tartaglia A., Locatelli M., Kabir A., Furton K.G., Macerola D., Sperandio E., Piccolantonio S., Ulusoy H.I., Maroni F., Bruni P. (2019). Comparison between Exhaustive and Equilibrium Extraction using different SPE Sorbents and Sol-gel Carbowax 20M Coated FPSE Media. Molecules.

[16] Malatesta L., Cosco D., Paolino D., Cilurzo F., Costa N., Di Tullio A., Fresta M., Celia C., Di Marzio L., Locatelli M. (2018). Simultaneous quantification of Gemcitabine and Irinotecan hydrochloride in rat plasma by using high performance liquid chromatography-diode array detector. J. Pharm. Biomed. Anal..

[17] Campestre C., Locatelli M., Guglielmi P., De Luca E., Bellagamba G., Menta S., Zengin G., Celia C., Di Marzio L., Carradori S. (2017). Analysis of imidazoles and triazoles in biological samples after MicroExtraction by Packed Sorbent. J. Enzyme Inhibit. Med. Chem..

[18] Locatelli M., Kabir A., Innosa D., Lopatriello T., Furton K.G. (2017). A Fabric Phase Sorptive Extraction-High Performance Liquid Chromatography-Photo Diode Array Detection Method for the Determination of Twelve Azole Antimicrobial Drug Residues in Human Plasma and Urine. J. Chromatogr. B.

[19] D’Angelo V., Tessari F., Bellagamba G., De Luca E., Cifelli R., Celia C., Primavera R., Di Francesco M., Paolino D., Di Marzio L. (2016). MicroExtraction by Packed Sorbent and HPLC-PDA quantification of multiple anti-inflammatory drugs and fluoroquinolones in human plasma and urine. J. Enzyme Inhibit. Med. Chem..

[20] Locatelli M., Ciavarella M.T., Paolino D., Celia C., Fiscarelli E., Ricciotti G., Pompilio A., Di Bonaventura G., Grande R., Zengin G. (2015). Determination of Ciprofloxacin and Levofloxacin in Human Sputum Collected from Cystic Fibrosis Patients using Microextraction by Packed Sorbent-High Performance Liquid Chromatography Photo Diode Array Detector. J. Chromatogr. A.

[21] Locatelli M., Cifelli R., Di Legge C., Barbacane R.C., Costa N., Fresta M., Celia C., Capolupo C., Di Marzio L. (2015). Simultaneous determination of Eperisone Hydrochloride and Paracetamol in mouse plasma by High Performance Liquid Chromatography-PhotoDiode Array Detector. J. Chromatogr. A.

[22] Locatelli M., Furton K.G., Tartaglia A., Sperandio E., Ulusoy H.I., Kabir A. (2019). An FPSE-HPLC-PDA method for rapid determination of solar UV filters in human whole blood, plasma and urine. J. Chromatogr. B.

[23] Locatelli M., Ferrone V., Cifelli R., Barbacane R.C., Carlucci G. (2014). Microextraction by packed sorbent and high-performance liquid chromatography determination of seven non-steroidal anti-inflammatory drugs in human plasma and urine. J. Chromatogr. A.

[24] Locatelli M., Tinari N., Grassadonia A., Tartaglia A., Macerola D., Piccolantonio S., Sperandio E., D’Ovidio C., Carradori S., Ulusoy H.I. (2018). FPSE-HPLC-DAD method for the quantification of anticancer drugs in human whole blood, plasma, and urine. J. Chromatogr. B.

[25] Kabir A., Furton K.G., Tinari N., Grossi L., Innosa D., Macerola D., Tartaglia A., Di Donato V., D’Ovidio C., Locatelli M. (2018). Fabric phase sorptive extraction-high performance liquid chromatography-photo diode array detection method for simultaneous monitoring of three inflammatory bowel disease treatment drugs in whole blood, plasma and urine. J. Chromatogr. B.

[26] Bielica-Daszkiewicz K., Voelkel A. (2009). Theoretical and experimental methods of determination of the breakthrough volume of SPE sorbents. Talanta.

[27] Bacalum E., Tanase A., David V. (2010). Retention mechanisms applied in solid phase extraction for some polar compounds. An. Univ. Bucar. Chim..

