# Supplementary material for: Novel MIPs-Parabens based SPE Stationary Phases Characterization and Application
Source: Molecules. 2019 Sep 13;24(18):3334. doi: 10.3390/molecules24183334 (PMC6767171; doi:10.3390/molecules24183334)
Supplement: Supplementary file 1 [file molecules-24-03334-s001.pdf]

Article

Novel MIPs-parabens based SPE stationary phases characterization and application

Angela Tartaglia [1], Abuzar Kabir [2,*], Songul Ulusoy [3], Halil Ibrahim Ulusoy [4], Giuseppe Maria Merone [5], Fabio Savini [6], Cristian D'Ovidio [7], Ugo de Grazia [8], Fabio Maroni [1], Pantaleone Bruni [1], Fausto Croce [1], Dora Melucci [9], Kenneth G. Furton [2], Marcello Locatelli [1,]**

[1] Department of Pharmacy, University of Chieti–Pescara "G. d'Annunzio", Via dei Vestini 31, Chieti 66100, Italy; angela.tartaglia@unich.it (A.T.); m.locatelli@unich.it (M.L.); fabio.maroni@unich.it (F.M.); pantaleonebruni@libero.it (P.B.) fausto.croce@unich.it (F.C.).

[2] Department of Chemistry and Biochemistry, International Forensic Research Institute, Florida International University, 11200 SW 8th St, Miami, FL 33199, USA; akabir@fiu.edu (A.K.); furtonk@fiu.edu (K.G.F.)

[3] Department of Chemistry, Faculty of Science, Cumhuriyet University, Sivas 58140, Turkey; sonulusoy@yahoo.com (S.U.);

[4] Department of Analytical Chemistry, Faculty of Pharmacy, Cumhuriyet University, Sivas 58140, Turkey; hiulusoy@yahoo.com (H.I.U.);

[5] Department of Neuroscience, Imaging and Clinical Sciences, University of Chieti–Pescara "G. d'Annunzio", Via dei Vestini 31, Chieti 66100, Italy; giuseppe.merone@unich.it (G.M.M.);

[6] Pharmatoxicology Laboratory - Hospital "Santo Spirito", Via Fonte Romana 8, Pescara 65124, Italy; fabio.savini@ausl.pe.it (F.S.);

[7] Department of Medicine and Aging Sciences, Section of Legal Medicine, University of Chieti–Pescara "G. d'Annunzio", Via dei Vestini 31, Chieti 66100, Italy; cristian.dovidio@unich.it (C.D.O.);

[8] IRCCS Neurological Institute Foundation Carlo Besta, Laboratory of Neurological Biochemistry and Neuropharmacology, Via Celoria 11, Milan 20133, Italy; ugo.degrazia@istituto-besta.it (U.D.G.);

[9] Department of Chemistry "G. Ciamician", University of Bologna, Via Selmi 2, Bologna 40126, Italy; dora.melucci@unibo.it (D.M.).

*Tel.: +1-305 348 2396; Fax: +1-305 348 4172; E-mail: akabir@fiu.edu (Abuzar Kabir)

**Tel.: +39-0871-3554590; Fax: +39-0871-3554911; E-mail: marcello.locatelli@unich.it (Marcello Locatelli)

Received: date; Accepted: date; Published: date

SUPPLEMENTARY MATERIAL

Section S.1

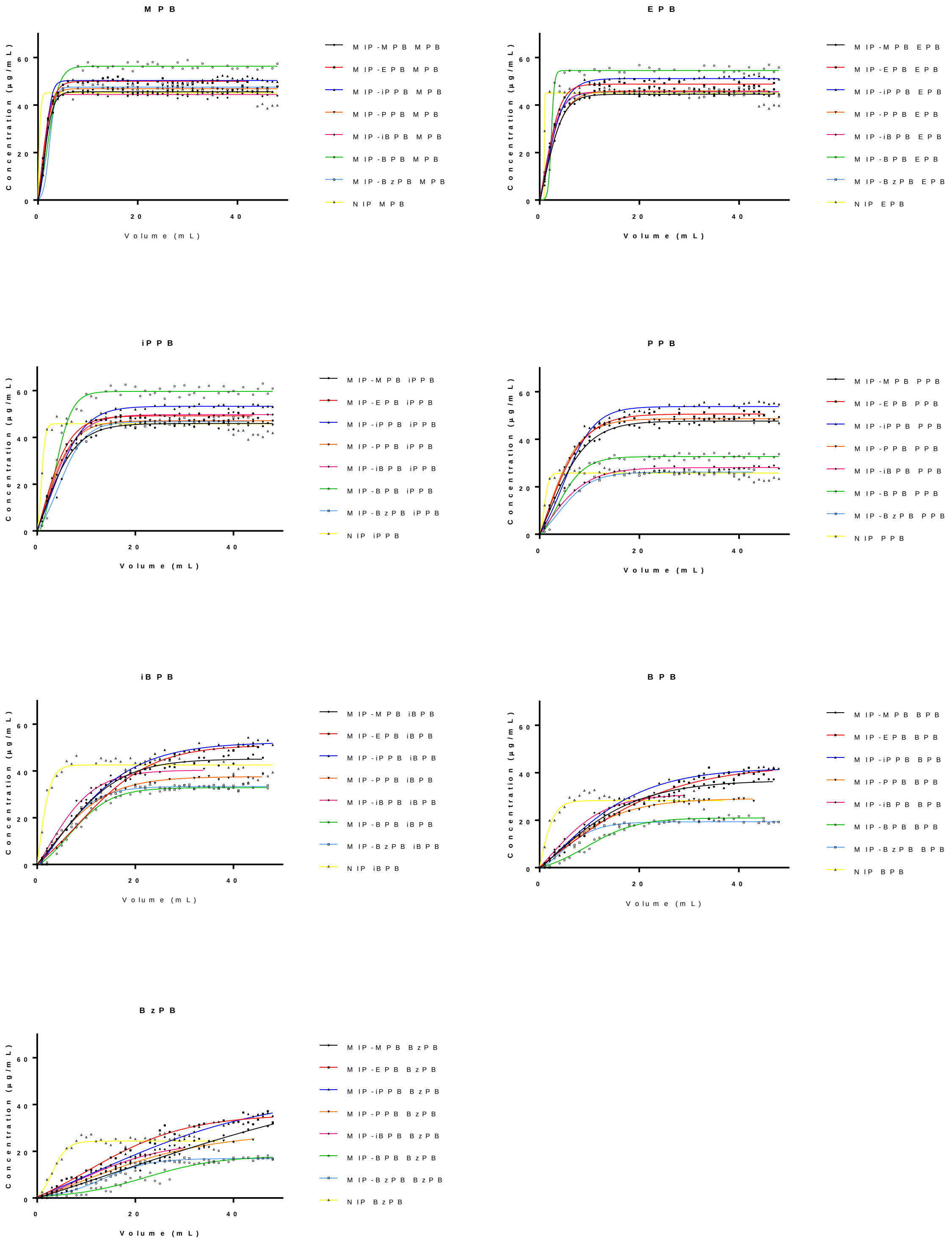

Figure S.1.1. Boltzmann's functions for single parabens.

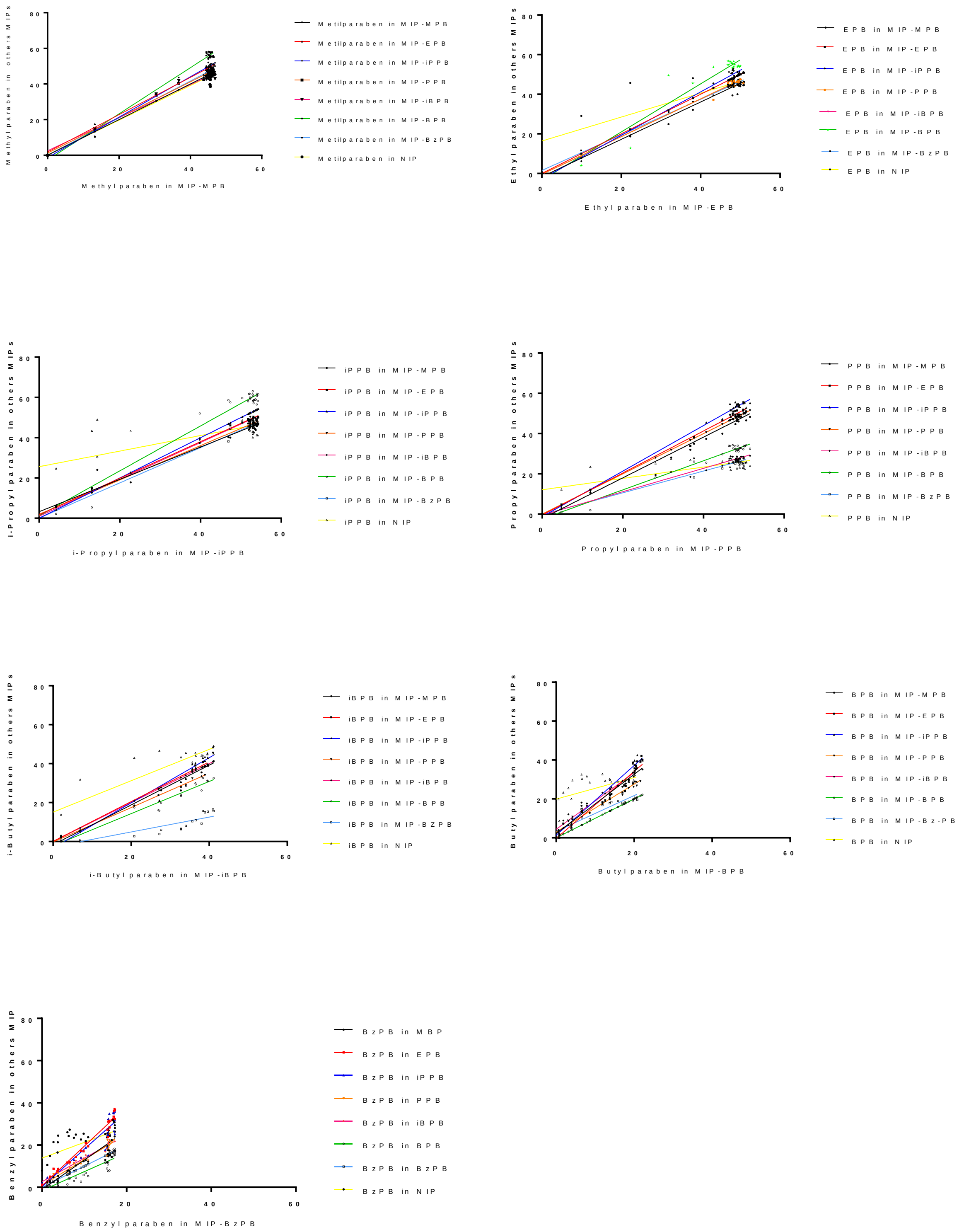

Figure S.1.2. Selectivity of each paraben. All the axes reports the concentration values (μg/mL).

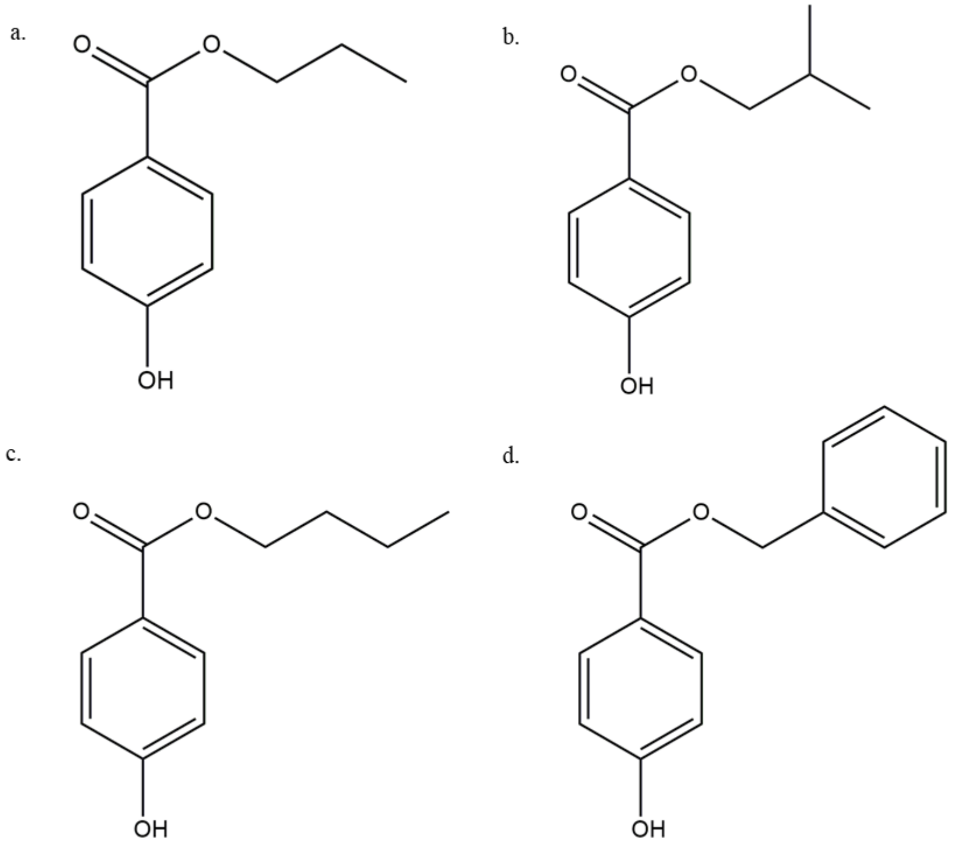

Figure S.1.3. Chemical structures of **a.** Propyl paraben; **b.** *iso*–Butyl paraben; **c.** Butyl paraben; **d.** Benzyl paraben

Section S.1.4. MIPs characterization

Thermogravimetric analysis was carried out on a series of MIP samples, on a Netzsch STA 2500 Regulus instrument. Measurements were conducted in air atmosphere in the 25°C-950°C temperature range.

Thermogravimetric plots (**Figure S.1.4.1.**) mainly consist in a series of pseudo-plateaus, indicating the occurrence of several physical and/or chemical processes with the increasing of temperature. The pseudo-plateau regions associated with weight loss have been respectively labelled from A to D. Region A (up to 270°C) could be assigned to the loss of physically adsorbed and volatile chemical species and solvent residues.

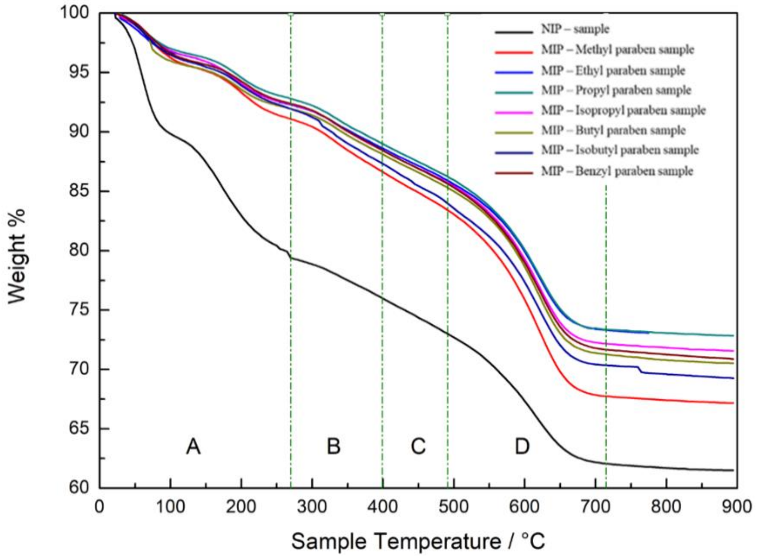

Figure S.1.4.1. Thermogravimetric (TGA) plots of the MIP sample series.

Looking at **Figure S.1.4.1.**, which reports the detailed regions of the MIP sample series TGA plots, region B and D, respectively located between 270°C÷399°C (region B) and 491°C÷715°C (region D), have been assigned to chemical processes. The 60% weight residue is related to the silica residue, which is the inorganic fraction of the sample. The reason for this assignment is clear from the investigation of the differential thermal analysis (DTA) plots of the MIP samples, reported in **Figure S.1.4.2**.

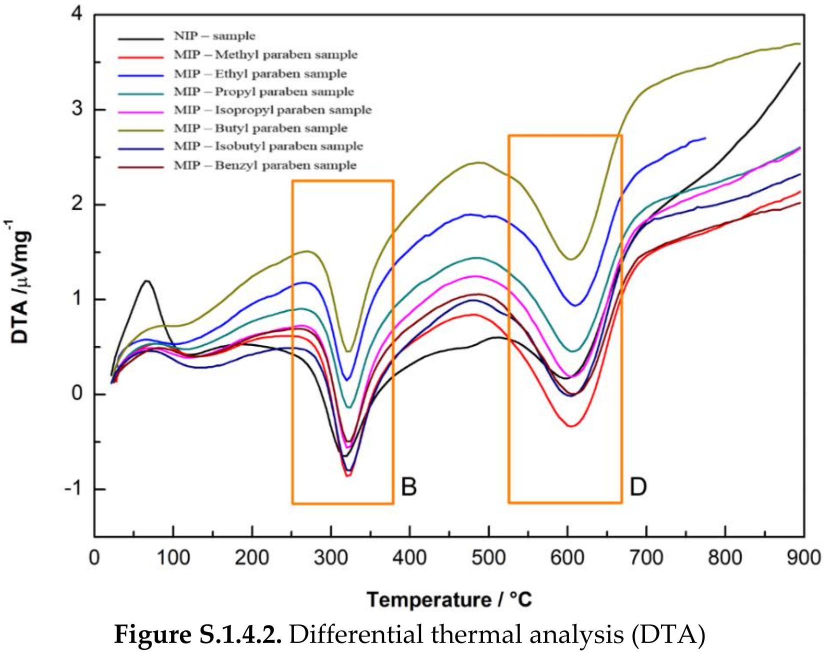

Figure S.1.4.2. Differential thermal analysis (DTA)

Looking at the DTA plots, region B and D are characterized by the presence of two exothermal peaks. Peak B appears to be quite sharp, and the partial decomposition of the functional moieties bonded to the Si atoms, as is expected from the MIPs synthetic procedure, can be hypothesized. On the other hand, region D

appears to be larger and falls in the temperature range in which the combustion reaction that generates CO_2 generally occurs, since the analysis has been conducted in air atmosphere. Given the mixed organic-inorganic nature of the presented MIPs, from both the thermogravimetric plot and the DTA analysis is possible to confirm a good reproducibility of the synthetic procedure.

FTIR spectra were recorded on a Perkin-Elmer Spectrum Two instrument, equipped with an Attenuated Total Reflectance (ATR) accessory, with a Zirconium Selenide (ZnSe) window. Spectra were recorded on pristine powder samples in the 4000 cm^{-1} - 450 cm^{-1} range. The Fourier transform infrared spectroscopy (FTIR) spectra band intensity has been normalized to 100% for all the eight samples. The preliminary FTIR analysis of the MIP samples, reported in **Figure S.1.4.3**, shows several frequency bands in the range of 3600 cm^{-1} - 3900 cm^{-1} (labelled as A), 2700 cm^{-1} - 3000 cm^{-1} (labelled as B), 1500 cm^{-1} - 2400 cm^{-1} (labelled as C) and below 1300 cm^{-1} (labelled as D).

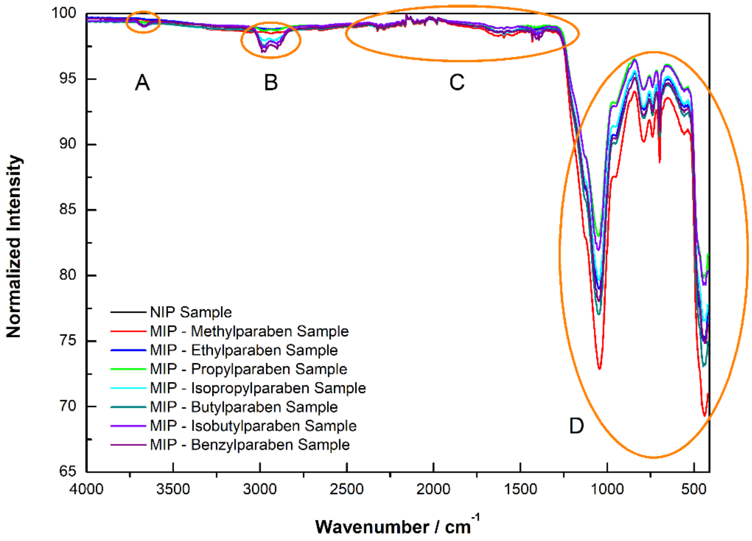

Figure S.1.4.3. Overall view of the MIP series FTIR spectra.

Broadly speaking, the spectra show a very good overlap in the various frequency ranges along all the sample series. In particular, as shown in **Figure S.1.4.4**, in both 2700 cm^{-1} - 3100 cm^{-1} and 3600 cm^{-1} - 3900 cm^{-1} frequency ranges, a progressive increase in intensity is clearly visible with the progressive increase in size and branching of the alkyl groups of the template molecule.

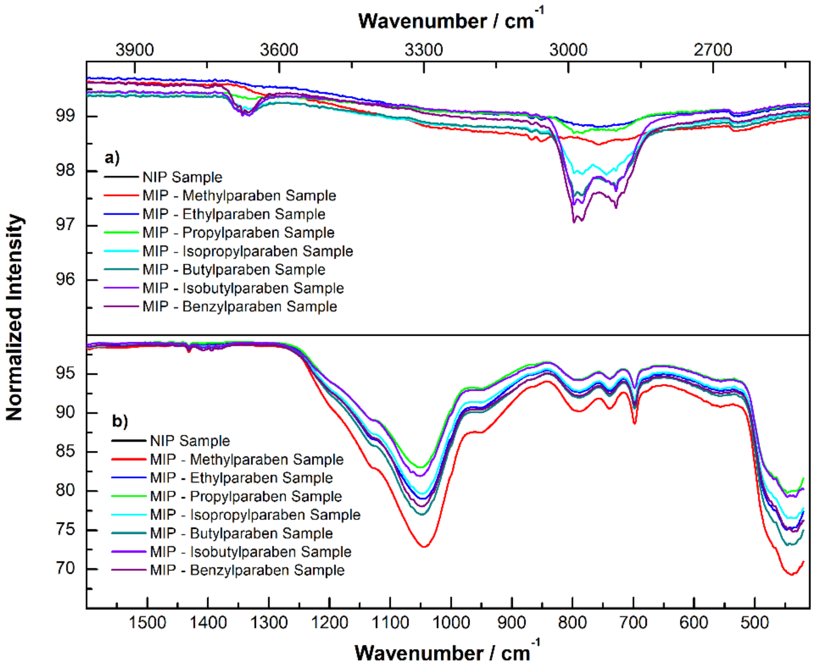

Figure S.1.*4.4*. Detail of a) 2600 cm^{-1} ÷ 4000 cm^{-1} frequency range and b) detail of 1500 cm^{-1} ÷ 410 cm^{-1} frequency range.

The absorption in the 3600 cm^{-1} - 3900 cm^{-1} range (region A) could be assigned to O-H bond stretching; since the bands appear to be relatively broad, it could be hypothesized that the -OH functional group is involved in some bonding interactions. On the other hand, the absorption bands in the 2700 cm^{-1} - 3100 cm^{-1} range (region B) could be assigned to C-H symmetric bond stretching. In particular, the increase in absorption intensity is mostly visible from the "MIP – Isopropyl paraben Sample". Then considering panel b of figure 6, the large peak cantered at 1045 cm^{-1} was assigned to the Si-O-Si bond stretching, while the series of peaks between 790 cm^{-1}-700 cm^{-1} was assigned to Si-C bond vibrations, being carbon atoms both aromatic and aliphatic. The reported data is consistent with the outcome of the synthetic procedure used for the preparation of the MIPs.
